# Reduced Risk of Sleep Disturbance and Obstructive Sleep Apnea Following Joint Replacement in Osteoarthritis Patients

**DOI:** 10.7150/ijms.116325

**Published:** 2025-10-20

**Authors:** Ying-Chi Fan, Shih-Wen Kao, Wei-Yang Lu, Chih-Hsin Tang, Jing-Yang Huang, Chia-Yi Lee, Chun-Yi Chuang, Shih-Chi Su, Shun-Fa Yang

**Affiliations:** 1 Institute of Medicine, Chung Shan Medical University, Taichung, Taiwan.; 2 Department of Neurology, Chung Shan Medical University Hospital, Taichung, Taiwan.; 3 School of Medicine, Chung Shan Medical University, Taichung, Taiwan.; 4 Department of Orthopedic Surgery, Chung Shan Medical University Hospital, Taichung, Taiwan.; 5 Department of Ophthalmology, Changhua Christian Hospital, Changhua, Taiwan.; 6 Department of Optometry, Chung Shan Medical University, Taichung, Taiwan.; 7 Department of Pharmacology, School of Medicine, China Medical University, Taichung, Taiwan.; 8 Department of Medical Laboratory Science and Biotechnology, Asia University, Taichung, Taiwan.; 9 Chinese Medicine Research Center, China Medical University, Taichung, Taiwan.; 10 Department of Medical Research, Chung Shan Medical University Hospital, Taichung, Taiwan.; 11 Department of Otolaryngology, Chung Shan Medical University Hospital, Taichung, Taiwan.; 12 Whole-Genome Research Core Laboratory of Human Diseases, Chang Gung Memorial Hospital, Keelung, Taiwan.; 13 Department of Medical Biotechnology and Laboratory Science, College of Medicine, Chang Gung University, Taoyuan, Taiwan.

**Keywords:** osteoarthritis, epidemiology, obstructive sleep apnea, sleep disturbance, surgery

## Abstract

Osteoarthritis (OA) is a prevalent condition characterized by inflammatory responses, and joint replacement surgery has been widely utilized for its management over the past several decades. Sleep disorders such as sleep disturbance and obstructive sleep apnea (OSA) are also associated with inflammation and pain. Therefore, the objective of this study was to investigate the association between joint replacement surgery and the subsequent risk of sleep disorders in individuals with OA. A retrospective cohort study was conducted using data from the TriNetX database. Individuals diagnosed with OA were categorized into two groups based on whether they underwent joint replacement surgery. A total of 135,607 individuals were included in both the surgery and non-surgery groups. The primary outcomes of interest were the incidence of sleep disturbance and OSA. A total of 19,146 episodes of sleep disturbance were observed in the surgery group, compared to 25,030 in the non-surgery group. Similarly, 8,715 cases of OSA occurred in the surgery group, whereas 11,472 were reported in the non-surgery group. Both sleep disturbance and OSA exhibited significantly lower incidence rates in the surgery group than in the non-surgery group (P < 0.001 for both). Consistently, cumulative incidence analyses also revealed significantly reduced rates of sleep disturbance and OSA in the surgery group (P < 0.001 for both). In conclusion, joint replacement surgery in patients with OA was associated with a decreased risk of subsequent sleep disorders, including both sleep disturbance and OSA.

## Introduction

Osteoarthritis (OA) is a degeneration change of joint that frequently develops in the knee and hip joints 1. Clinical manifestations of OA contain the fluctuating morning stiffness, crepitus on motion and intermittent or permanent joint pain 2. Severe OA can prominently diminish the daily activity and the joint replacement surgeries involving the total knee arthroplasty (TKA) would be arranged to reduce the illness 3-5. Outcomes of TKA and other joint replacement surgeries are generally sub-optimal while the persistent pain after the surgery could still negatively impact the quality of life of the OA population 6, 7.

Relationship between OA and certain diseases were illustrated in the preceding compositions 8. OA presence is significantly correlated to the vascular disease and the potential premature death 9. Besides, diabetes mellitus would contribute to higher likelihood of OA development compared to the non-diabetes mellitus patients 10-12. Except the above two diseases, the obesity status is significantly correlated to OA occurrence involving the knee and hip subtypes 13. About the joint replacement surgery, the venous thromboembolism may occur after the receipt of TKA or hip replacement surgery 14.

Sleep disturbance refers to any disruption of normal sleep patterns and is often associated with daytime drowsiness and an increased risk of neurodegenerative diseases 15, 16. In contrast, obstructive sleep apnea (OSA) is characterized by snoring, repeated episodes of breathing cessation during sleep, and excessive daytime sleepiness 17. Previous studies have shown that both sleep disturbances and OSA are associated with systemic conditions such as hypertension and chronic pain 15, 18-20. Regarding the relationship between OA and the sleep disorders, OA is frequently accompanied by symptoms such as chronic pain, compromised mental well-being, and disturbances in sleep 21-23. Furthermore, a strong association has been identified between OA and OSA, with evidence suggesting that both conditions may share common inflammatory mechanisms 24, 25. Although sleep disturbances are commonly observed in patients with osteoarthritis (OA) undergoing joint replacement surgery 26, 27, surgical intervention may help reduce the severity of these disturbances 28. However, the relationship, or possible interaction, among OA, joint replacement surgery, and the two sleep disorders had not been fully investigated. Because the OA could associate with pain and hypertension 2, 29, the management of OA may alter the chance of developing sleep disorders in the OA population which need validation.

The objective of this study was to investigate the potential relationship between undergoing joint replacement surgery and the subsequent risk of sleep disorders in patients with OA. In addition, the incidence of sleep disturbance and OSA was evaluated across OA populations with varying characteristics.

## Materials and Methods

### Data source

This study adhered to the declaration of Helsinki in the 1964 and the sequential amendments. In addition, this study was agreed by the Institute Review Board of Chung Shan Medical University (project code: CS2-23208). TriNetX database is a dataset that restores the claimed data from numerous bunches of medical insurance foundations in the United States. The total individual numbers accessible in the TriNetX database is more than 200 million individuals. Medical documents accessible in the TriNetX database contain the International Classification of Diseases, Tenth Revision, Clinical Modification (ICD-10-CM) codes, sex, educational level, ethnicity, age, socioeconomic status, medical encounter condition, the image test codes, the laboratory test codes, the laboratory test results, the surgery codes, the procedure codes, and finally the Anatomical Therapeutic Chemical (ATC) codes for the prescriptions.

### Individual selection

**Figure 1:**
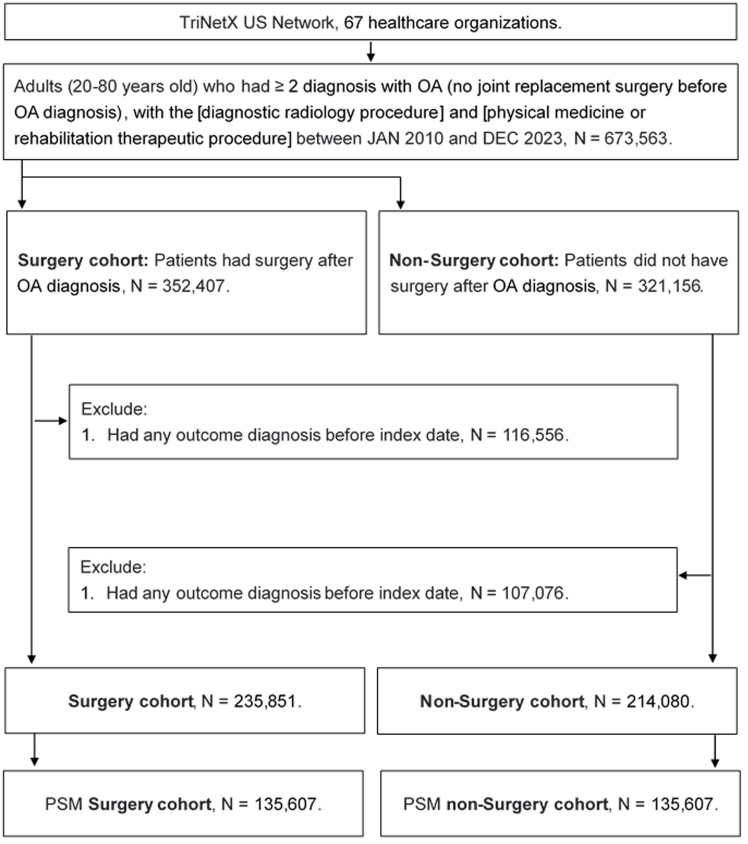
The flowchart of participant selection. Abbreviations: N: number, OA: osteoarthritis, PSM: propensity score matching.

A retrospective cohort study was conducted, and individuals were identified as having OA if they met the following criteria: (1) a diagnosis of OA based on relevant ICD-10-CM codes; (2) receipt of a complete blood cell count, white blood cell differential count, and X-ray examination prior to the OA diagnosis; and (3) age between 20 and 80 years. Individuals were excluded if they (1) had experienced the primary outcome (described in a later section), or (2) had undergone joint replacement surgery within six months prior to the index date. Subsequently, each OA patient who underwent joint replacement surgery was matched to an OA patient who did not undergo the surgery, using propensity score matching (PSM). The PSM approach collected the demography, systemic morbidities, laboratory results and medicines into the specific score system and then match different individuals. Finally, a total of 135,607 and 135,607 individuals were collected into the surgery and non-surgery groups, respectively. The flowchart of individual selection is exhibited in Figure 1.

### Primary outcome

The primary outcomes of this study were the onset of sleep disorders, specifically sleep disturbance and OSA. In this study, sleep disturbance was defined as the presence of at least one documented diagnosis of a sleep-related disorder, identified using ICD-10-CM codes during the designated observation period. Specifically, diagnoses classified under ICD-10-CM codes F51.x (nonorganic sleep disorders, such as insomnia not caused by a medical condition) and G47.x (organic sleep disorders, including insomnia, hypersomnia, circadian rhythm disorders, and others) were included. OSA was defined based on two sequential criteria: (1) a documented diagnosis of OSA according to the corresponding ICD-10-CM codes, and (2) the performance of a polysomnography (sleep study), identified using relevant procedural codes, prior to the diagnosis. Only sleep disorder events that occurred after the index date were considered as primary outcomes in this study. The index date (start of outcome collection) was determined at 6 months after the diagnosis of OA for non-surgery group, and 6 months after the joint replacement surgery in the surgery group. All the individuals in this study were pursued to the outcome competence, individual withdraw from their health insurance program or the deadline of TriNetX database: December, 31, 2023.

### Confounder selection

To better examine the connection between OA with joint replacement surgery and sequential sleep disorder, the effect of the sequential confounding factors were adjusted in multivariable analysis: age, race, sex, medical encounter, Socioeconomic and psychosocial defect, diabetes mellitus, hypertension, peripheral vascular disease, cerebrovascular disease, dyslipidemia, chronic lower respiratory diseases, kidney disease, alcohol related disorders, nicotine dependence, estimated glomerular filtration rate (eGFR), body mass index (BMI), glycated hemoglobin (HbA1c), serum leukocyte, LDL, HDL, and serum cholesterol. The emergences of above confounding factors agree with the demographic codes, ICD-10 CM codes, procedure codes and ATC codes. To ensure the presence of systemic co-morbidities is long enough to change the possibility of diseases, only those systemic diseases that proceed for longer than two years were taken into the statistic model.

### Statistical analysis

The SAS version 9.4 (SAS Institute Inc, Cary, NC, USA) was managed for statistical analyses in this study. The descriptive analysis was implemented to demonstrate the basic conditions between the surgery and the non-surgery groups, and the standard mean difference (SMD) was implemented to examine the difference of each data between the surgery group and non-surgery group. A SMD larger than 0.1 was defined as significant difference in this study. After that, the Cox proportional hazard regression was implemented to evaluate the incidence of the sleep disorders between the two groups, and the adjusted hazard ratio (aHR) with its 95% confidence interval (CI) for the sleep disorder occurrence was created. The race, sex, age, medical encounter, systemic morbidities, lifestyle factors, and laboratory results which mentioned in above section were inserted into the Cox proportional hazard regression to weigh their influence on the sleep disorders occurrence. After that, the Kaplan-Meier curve was made and the cumulative incidence of sleep disorders between the surgery group and non-surgery group were created by the log-rank test. For sensitive analysis, the individuals were distributed into different subgroup in agreement with race, age, sex, eGFR, LDL, HDL, and HbA1c, and Cox proportional hazard regression was implemented again to investigate the primary outcomes in different populations. Statistical significance was determined as P < 0.05 and the P value interior to 0.001 was displayed as P < 0.001.

## Results

Basic conditions of the surgery group and non-surgery groups are demonstrated in Table 1. The mean age was 65.0±10.6 and 65.0±12.0 years in the surgery and non-surgery group, respectively. The age, race, and sex distributions were similar between the two groups due to the PSM process (all SMD < 0.1). About the systemic diseases and lifestyle factors, the values also illustrated non-significant differences between the two groups (all SMD < 0.1). For the laboratory data, the surgery and non-surgery groups demonstrated similar values of all laboratory data (all SMD < 0.1), except the HDL value was significantly higher in the surgery group than the non-surgery group (SMD = 0.1239) (Table 1).

**Figure 2:**
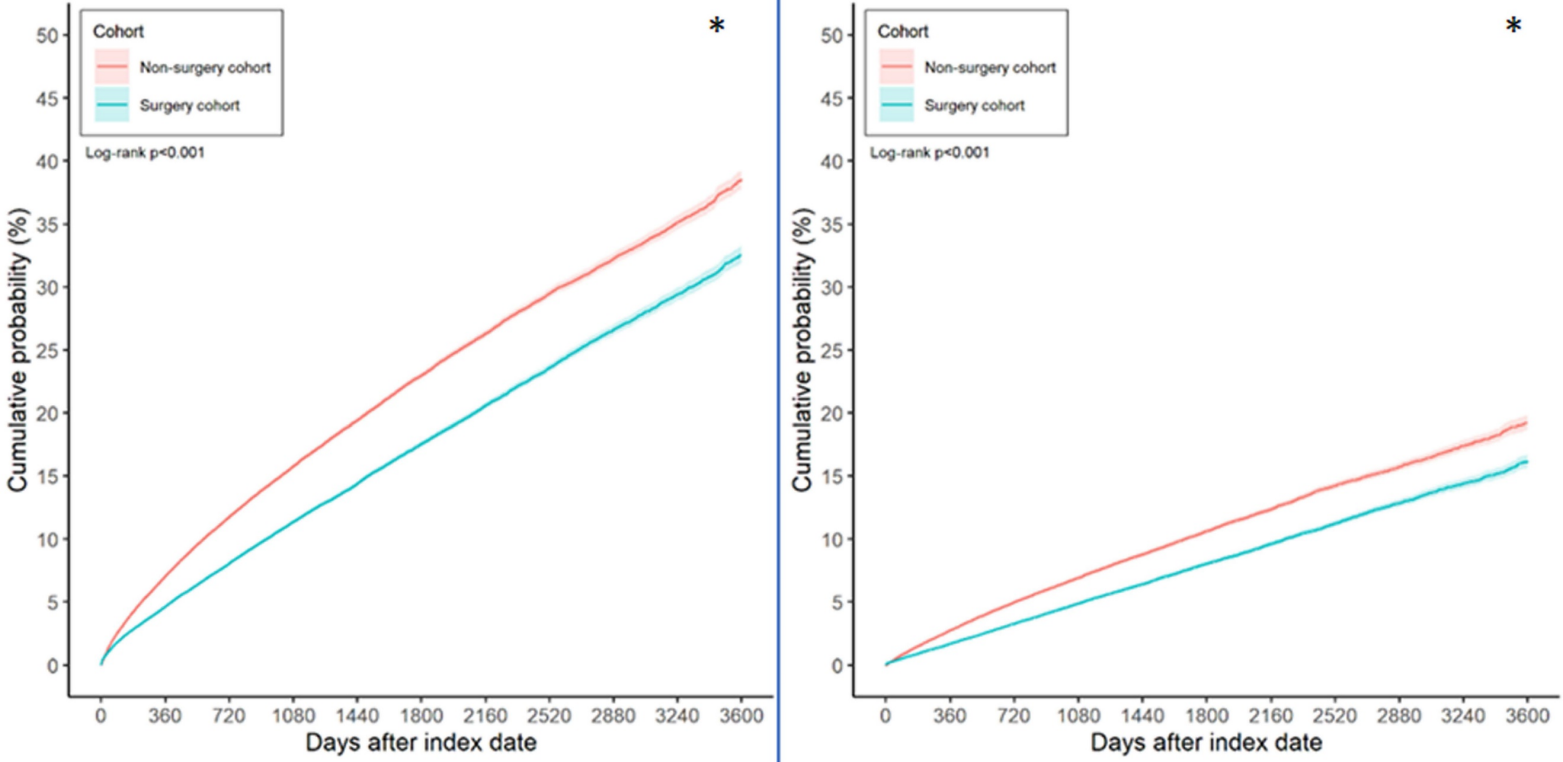
The Kaplan-Meier curve of sleep disorders between groups. (A) The cumulative incidence of sleep disturbance between groups. (B) The cumulative incidence of obstructive sleep apnea between groups. The symbol * denotes significant difference between groups.

After the follow up period ranged from 1 to 12 years, there were 19,146 and 25,030 sleep disturbance episodes occurred in the surgery and non-surgery groups, respectively. Besides, there were 8,715 and 11,472 OSA events developed in the surgery and non-surgery groups, respectively. After balancing all confounding factors, the sleep disturbance (aHR: 0.738, 95% CI: 0.724-0.752, P < 0.001) and OSA (aHR: 0.744, 95% CI: 0.724-0.765, P < 0.001) demonstrated a significantly lower incidences in the surgery group compared to the non-surgery group. The cumulative incidence of sleep disturbance and OSA are presented in Figure 2, and the cumulative incidence of sleep disturbance and OSA were significantly lower in the surgery group compared to the non-surgery group (both P < 0.001) (Figure 2).

**Figure 3:**
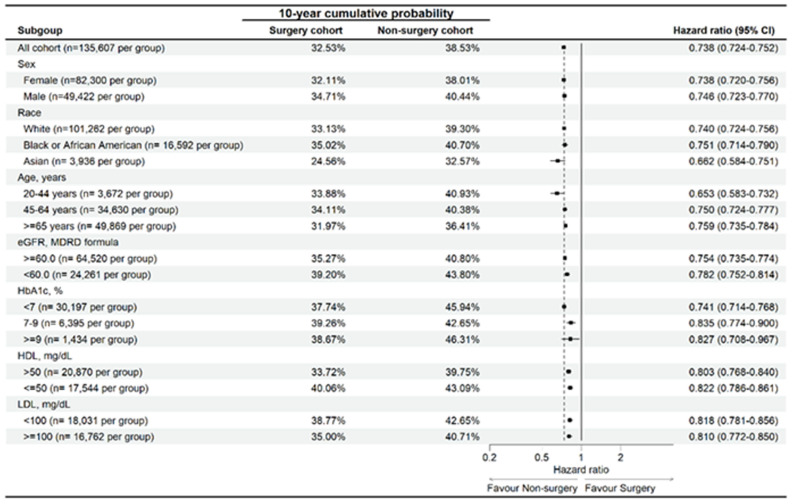
Risk of sleep disturbance in participants with osteoarthritis stratified by age, sex, race, eGFR, HDL, LDL, and HbA1c. Abbreviations: eGFR: estimated glomerular filtration rate, HbA1c: glycated hemoglobin, N: number.

**Figure 4:**
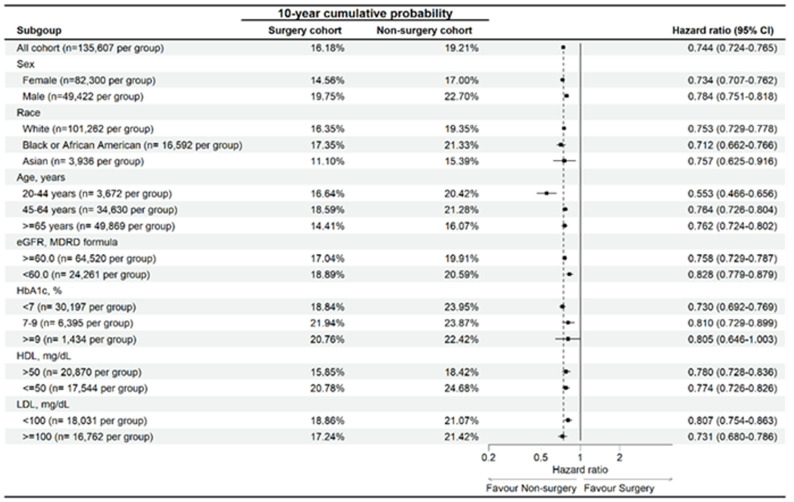
Risk of obstructive sleep apnea in participants with osteoarthritis stratified by age, sex, race, eGFR, HDL, LDL, and HbA1c. Abbreviations: eGFR: estimated glomerular filtration rate, HbA1c: glycated hemoglobin, N: number.

In the sensitive analysis, surgery populations with different characteristics (old age, female sex, African American, impaired renal function, hyperglycemia, dyslipidemia) represented significantly lower risk of developing sleep disturbance than the non-surgery population (Figure 3). Similarly, the surgery populations with different characteristics (same as above) represented significantly lower risk of developing OSA than the non-surgery population except for those with HbA1c level more than 9 (aHR: 0.805, 95% CI: 0.646-1.003) (Figure 4).

## Discussion

In this study, incidences of sleep disorders including sleep disturbance and OSA were significantly lower in the OA patients received joint replacement surgery than the OA patients did not receive surgery. Moreover, the cumulative incidences of the sleep disorders were significantly lower in the OA patients received joint replacement surgery. On the other hand, the surgery populations with different characteristics illustrated lower risk of occurring sleep disorders than the non-surgery population while the high HbA1c population showed similar rate of OSA between the surgery and non-surgery groups.

Development of OA is related to several pathophysiology and co-morbidities in accordance with earlier literatures 8, 10, 30, 31. The mechanical pressure and sequential inflammation count for the major pathophysiology of OA presence in which the low-extent chronic inflammation in the joint space was discovered in the OA cases 32, 33. In other literatures, the prolonged inflammation and neutrophil aggregation could insult the joint tissue and contribute to the OA development 34, 35. In addition, the high expressions of inflammatory biomarkers involve the prostaglandins and interleukin were discovered in the people with OA 30, 36. In addition to inflammatory reaction, the enhancement of oxidative stress, subchondral bone remodeling, and osteophyte formation were discovered during the OA formation 32, 37. Concerning the OA and other morbidities, the OA occurrence is associated with the attendance of glucose intolerance, diabetes mellitus and hyperlipidemia 8, 38, 39. Also, the coronary heart disease is related to higher risk of OA and the symptoms of coronary heart disease can be exaggerated by the OA attendance 40, 41. The sleep disturbance can be featured with the elevation of inflammatory markers like interleukin and C-reactive protein 15, 42. Also, the diseases with chronic pain or irritation can correlated to the development of sleep disturbance 43, 44. On the other side, the OSA is associated with increased sympathetic tone and inflammation biomarkers 45-47, and several diseases with same characters like the hypertension and the coronary heart disease relate to the OSA occurrence 17, 48, 49. Furthermore, the presence of chest pain is significant higher in the population with OSA 50. The OA and sleep disorders share similar pathophysiology 15, 34, 49, and the pain cause by OA may be relief after the surgery and the pain is related to sleep disorders 18, 20. Thus, we speculate that the arrangement of surgery in OA patient may correlate to lower risk of sleep disorder, which is supported by the results of this study.

The arrangement of joint replacement surgery is associated with the lower incidences of sleep disturbance and OSA. Previous studies have extensively documented sleep disturbances or insomnia following joint replacement surgeries 51-56. For example, a systematic review reported that sleep disturbances are common during the early stages of recovery after TKA and may be related to pain. However, sleep quality tends to improve and pain intensity decreases after three months 54.

To our knowledge, this study may be a preliminary experience to represent the significant correlation between the arrangement of joint replacement surgery and the lower incidence of sleep disturbance and OSA in OA population. In addition, we excluded sleep disturbance and OSA episodes recorded prior to the OA presence, thus the time sequence between the joint replacement surgery and sleep disorder events may be constructed. Furthermore, we balanced several confounders of sleep disorders including age, sex, hypertension and heart disease into the Cox proportional hazard regression 16, 17, 48, 57. Besides, the laboratory results were also considered which can reflect the severity of diseases like the diabetes mellitus and dyslipidemia. As a result, the arrangement of joint replacement surgery may be an independent protective factor for sleep disorder in OA patients. The previous researches illustrated the reduction of depression and anxiety in the patients with OA and received joint replacement surgery 58. Consequently, the sleep quality, which is also a psychological disorder partially 59, may be improved after the surgery to remove the pain and inflammation of OA. On the other side, the cumulative probabilities of sleep disturbance and OSA were significantly lower in the OA patients received joint replacement surgery, which may indicate the risk of sleep disorders would gradually increase in the OA patients without surgery compared to the OA patients with surgery. This finding further confirmed the relationship between surgical management and lower risk of sleep disorder in OA.

In the sensitive analysis, OA patients with joint replacement surgery and different characters showed lower risk of developing sleep disturbance than the OA patients without surgery. Also, the OA patients with joint replacement surgery and different characters showed lower risk of developing OSA than the OA patients without surgery. The above findings further illustrated the universal influence of joint replacement surgery for sleep disorders in OA patients with different features. The only exception is the high HbA1c population, in which the incidences of OSA were similar between the surgery group and non-surgery group. There was rare study to illustrate this phenomenon. In the previous studies, the high HbA1c value may be a predisposing factor for the OSA since it can be served as the severity of diabetes mellitus which is a risk factor for OSA 17, 60. Accordingly, the risk of developing OSA in the patients with high HbA1c concentration may become similar in the non-surgery population and surgery population since the baseline possibility of OSA occurrence were elevated in such population. Nevertheless, the patient numbers of the high HbA1c concentration were relative few in which only about 1 percent of individuals of both groups were enrolled in such population. Perhaps another study with sufficient patients with high HbA1c status is advocated to confirm this results.

Respecting the epidemiological circumstances, OA is a prevalent disorder in many regions according to previous study 2. It is estimated that about 300 million people is suffered from hip OA and knee OA worldwide based on previous article 61, and there was 19% adult was found to have radiographic knee OA 13. Moreover, the OA is a leading etiology of motion disability in many regions especially the knee OA 62. For the people with advanced OA, the joint replacement surgery is frequently recommended which results in overwhelming medical cost 29. In fact, the TKA is by far the most common joint replacement approach which operated in above 60 thousand individuals in the England 6. On the other side, the sleep disorders are also a common disease throughout the world 19, 44, 63. There are about half of adults older than 60 years report the sleep disturbance 63, and the prevalence of OSA is above 10 percent in different populations 60, 64. Because both the OA and sleep disorder affect large proportion of people, any correlation between them and their management should be represented.

This study has several limitations. First, the TriNetX database contains only claims data and diagnostic codes, excluding detailed laboratory results. Consequently, critical information such as the exact anatomical site and severity of OA, radiographic findings, patient-reported pain scores, surgical details and perioperative conditions of joint replacement surgery, surgical outcomes, the degree and quality of sleep disorders, polysomnography results for the OSA population, treatments and clinical outcomes of sleep disorders, symptom improvement, pain and functional scores during follow-up, recurrence of sleep disorders, and detailed comorbidity profiles were not accessible. Second, the retrospective cohort design inherent to database studies may limit the homogeneity of the study population compared to a prospective design, despite the application of PSM to mitigate this issue. Third, some patients with sleep disorders may have sought alternative treatments such as behavioral therapy or traditional Chinese medicine, potentially leading to underestimation of the true incidence of sleep disorders. Finally, although we hypothesize that postoperative improvements in pain and inflammation contribute to better sleep outcomes, we were unable to directly assess these mechanisms. The TriNetX platform does not consistently provide standardized pain assessments (e.g., visual analog scale), inflammatory biomarkers (e.g., C-reactive protein, interleukins), or objective sleep measures such as polysomnography or actigraphy. Therefore, our analysis was limited to diagnostic codes and prescription data, which may not fully capture the clinical complexity of pain, inflammation, or sleep quality.

In conclusion, the arrangement of joint replacement surgery is related to lower risk of sleep disturbance and OSA developments in OA population after considering multiple confounders. Furthermore, the lower incidence of sleep disorders in OA patients received surgery become more prominent as the OA period increase. Consequently, joint replacement surgery might be suggested to those patients with known risk factor for sleep disorders and have indication of joint replacement surgery. Further large-scale prospective study to evaluate the possible role of pain and functionality on sleep disorder risks in OA patients with or without joint replacement surgery and the correlation between joint replacement surgery and the therapeutic outcomes of sleep disorders in OA patients is mandatory.

## Figures and Tables

**Table 1:** Baseline characteristics among Surgery cohort and Non-Surgery cohort before and after propensity score matching.

Characteristics	Surgery cohort	Non-Surgery cohort	SMD
N	135607	135607	
Age at Index	65.0±10.6	65.0±12.0	0.0024
Sex			
Female	81457 (60.1%)	81155 (59.8%)	0.0045
Male	49266 (36.3%)	49554 (36.5%)	0.0044
Race			
White	99834 (73.6%)	99460 (73.3%)	0.0062
Black or African American	16812 (12.4%)	17356 (12.8%)	0.0121
Asian	4472 (3.3%)	4427 (3.3%)	0.0019
Socioeconomic and psychosocial defect	1185 (0.9%)	1342 (1.0%)	0.0121
Medical encounter			
Preventive Medicine Services	8141 (6.0%)	8293 (6.1%)	0.0047
Inpatient Encounter	77047 (56.8%)	39544 (29.2%)	0.5818
Emergency	18359 (13.5%)	20019 (14.8%)	0.0351
Critical Care Services	1183 (0.9%)	5799 (4.3%)	0.2162
Rehabilitation Therapeutic Procedures	62790 (46.3%)	135607 (100.0%)	1.4139
Lifestyle			
Nicotine dependence	19043 (14.0%)	19229 (14.2%)	0.0039
Alcohol related disorders	2918 (2.2%)	3067 (2.3%)	0.0075
Comorbidities			
Hypertension	72282 (53.3%)	72867 (53.7%)	0.0086
Dyslipidemia	57783 (42.6%)	57810 (42.6%)	0.0004
Ischemic heart diseases	16099 (11.9%)	16569 (12.2%)	0.0106
Diabetes mellitus	24652 (18.2%)	25263 (18.6%)	0.0116
Kidney disease	13663 (10.1%)	14532 (10.7%)	0.0210
Peripheral vascular disease	9821 (7.2%)	10340 (7.6%)	0.0146
Chronic lower respiratory diseases	19585 (14.4%)	19959 (14.7%)	0.0078
Cerebrovascular diseases	6073 (4.5%)	6581 (4.9%)	0.0178
Lab data			
BMI	30.0±6.1	30.0±7.2	0.0029
eGFR	78.5±23.4	77.7±27.7	0.0274
Leukocytes in Blood	7.5±21.2	8.4±29.1	0.0349
Plasma cholesterol	182.3±45.5	178.5±49.5	0.0812
HDL	52.3±22.0	49.6±22.1	0.1239*
LDL	102.0±37.3	100.7±38.9	0.0339
HbA1c	6.1±1.1	6.4±1.6	0.2245

BMI: body mass index, eGFR: estimated glomerular filtration rate, HbA1c: glycated hemoglobin, N: number, SMD: standard mean difference * denotes significant difference between groups

**Table 2:** Primary outcomes between the two groups.

Study event	N	Cumulative probability				aHR (95% CI)	P value
		1-year	3-year	5-years	10-years		
Sleep disturbance							
Surgery cohort	19,146	4.66%	11.38%	17.56%	32.53%	0.738 (0.724-0.752)	<0.001*
Non-surgery cohort	25,030	7.07%	15.79%	22.97%	38.53%	Reference	
OSA							
Surgery cohort	8,715	1.69%	4.90%	8.05%	16.18%	0.744 (0.724-0.765)	<0.001*
Non-surgery cohort	11,472	2.75%	6.92%	10.63%	19.21%	Reference	

aHR: adjusted hazard ratio, CI: confidence interval, N: number, OSA: obstructive sleep apnea * denotes significant difference between groups
